# Nrf2 deletion causes “benign” simple steatosis to develop into nonalcoholic steatohepatitis in mice fed a high-fat diet

**DOI:** 10.1186/1476-511X-12-165

**Published:** 2013-11-04

**Authors:** Chunhua Wang, Yizhe Cui, Chunyan Li, Yanhua Zhang, Shang Xu, Xiaochong Li, Hong Li, Xiuying Zhang

**Affiliations:** 1 Department of Basic Veterinary Science, College of Veterinary Medicine Northeast Agricultural University, Harbin, Heilongjiang 150030, China

**Keywords:** Nrf2-null, High fat diet, SS, NASH, Fatty acid

## Abstract

**Background:**

Nonalcoholic fatty liver disease begins with the aberrant accumulation of triglyceride in the liver. Its spectrum includes the earliest stage of hepatic simple steatosis (SS), nonalcoholic steatohepatitis (NASH), cirrhosis, and hepatocellular carcinoma. Generally, hepatic SS is often self-limited; however 10%-30% of patients with hepatic SS progress to NASH. The cause(s) of the transition from SS to NASH are unclear. We aimed to test the contribution of nuclear erythroid 2-related factor 2 (Nrf2) on the progression of “benign” SS to NASH in mice fed a high fat diet. In doing so, we discovered the influence of fatty acid in that progression.

**Method:**

The involvement of Nrf2 in defending against the development of NASH was studied in an experimental model induced by a high-fat diet. Wild-type and Nrf2-null mice were fed the diet. Their specimens were analyzed for pathology as well as for fatty acid content and ratios.

**Result:**

In feeding the high-fat diet to the Wild-type and the Nrf2-null mice, the Wild-type mice increased hepatic fat deposition without inflammation or fibrosis (i.e., simple steatosis), while the Nrf2-null mice had significantly more hepatic steatosis and substantial inflammation, (i.e., nonalcoholic steatohepatitis). In addition, as a result of the high-fat diet, SFA (C20: 0, C22: 0) and MUFA (C18: 1, C20: 1) content in Nrf2-null mice were significantly higher than in Wild-type mice. In the Nrf2-null mice the PUFA/TFA ratio decreased; conversely, the MUFA/TFA ratio increased.

**Conclusion:**

The deletion of Nrf2 causes “benign” SS to develop into NASH in mice fed with a high-fat diet, through prompt fatty acid accumulation and disruption of hepatic fatty acid composition in the liver.

## Introduction

Nonalcoholic fatty liver disease (NAFLD) is a burgeoning health problem that affects one-third of adults and an increasing number of children in developed countries. The disease begins with the aberrant accumulation of triglyceride (TG) in the liver, which in some individuals elicits an inflammatory response that can progress to cirrhosis and liver cancer [1].

The earliest stage of NAFLD is hepatic steatosis, which is characterized by the deposition of TG as lipid droplets in the cytoplasm of hepatocytes, and the presence of cytoplasmic TG droplets in more than 5% of hepatocytes [2]. The hepatic steatosis is often limited. However, approximately 10%-30% of patients with hepatic steatosis develop nonalchoholic steatohepatitis (NASH) [3], prevalent among a largely middle-aged population and distinguished from SS by the presence of hepatocyte ballooning, cell death, inflammation, and/or fibrosis. Subsequently, between 10 and 29% of patients with NASH progress to cirrhosis [4] and 4 to 27% of patients with NASH-induced cirrhosis develop hepatocellular carcinoma [5]. NASH is the key stage in the progression of NAFLD but what causes the transition from SS to NASH is unclear.

Nuclear erythroid 2-related factor 2 (Nrf2) is a significant transcription factor for the induction of a variety of detoxification enzymes, biotransformation enzymes, and xenobiotic efflux transporters, which can regulate up or down inflammatory cytokine genes, fibrogenesis-related genes, and fatty acid metabolism via the Nrf2 transcriptional pathway. It has been proposed that the Nrf2 gene plays a role in NASH because deleting it from mice results in rapid onset and progression of the disease [6].

However, whether Nrf2 plays a role in the transition from SS to NASH induced by a high-fat diet (HFD) has been unclear. We hypothesize that Nrf2 does play a role and that deletion of Nrf2 will make SS develop into NASH induced by HFD in the short term. To test the hypothesis, we have analyzed and evaluated biochemical and pathological characteristics and the expression levels of genes involved in inflammatory cytokine in the livers of wild-type (WT) and Nrf2-null mice that are placed on a HFD, a proven method of stimulating NAFLD in rodents [7]. Moreover, the studies test the content and ratio of fatty acids in the liver, in order to explore the function of fatty acid in the progression. By using Nrf2-null mice fed an HFD, we first demonstrated that Nrf2 deletion led to the development of NASH from SS, a result that was impossible to produce in the WT induced by HFD over a period of four weeks. The contents of SFA (C20: 0, C22: 0) and MUFA (C18: 1, C20: 1) increased, and the ratios of PUFA and MUFA in SFA homeostasis are important in bringing about the progression of SS to NASH.

## Materials and methods

### Animals and experimental design

WT and Nrf2-null mice, all 8-week-old male with ICR background were fed an HFD [8] or control diet for 4 weeks. At the end of the experiment, serum and liver tissue specimens were collected for analysis. All experiments were performed under protocols approved by the Institutional Animal Care and Use Committees of Northeast Agricultural University.

### Histological analysis

Sections (approx 4 μm) of formalin-fixed and paraffin-embedded liver tissue were stained with hematoxylin and eosin before being examined under a light microscope. Scoring for the degree of steatosis, inflammation, and fibrosis in the livers of all mice was carried out by a pathologist who was unaware of the genotypes of the mice and whether they had been fed the HFD or control diet.

### Biochemical analysis

Blood specimens were collected immediately before sacrifice after a 4-hour fast and tested for plasma alanine aminotransferase (ALT), cholesterol (TC), total triglyceride (TG), aspartate transminase (AST), alkaline (ALP), glucose (GLU), and very low-density lipoprotein (VLDL), which were determined using a Beckman CX4 automatic biochemical analyzer (Beckman Coulter, Inc., USA).

### Lipid analysis

Measurement of the fatty acid content of the liver specimens was carried out using gas chromatography mass spectrometry (GC-MS) (HP6890N-5973 Agilent, Hewlett-Packard, USA) [8].

### Real-time quantitative polymerase chain reaction

Total RNA was isolated from whole liver tissue using RNeasy Mini Columns (Qiagen, Valencia, CA) and reverse transcribed to complementary DNA. Quantitative real-time reverse-transcription polymerase chain reaction (RT-PCR) was performed using the ABI 7500 sequence detection system. Assays were performed in triplicate, and results were expressed as relative gene expression normalized to expression levels of housekeeping gene (GAPDH).

### Statistical analysis

Results were expressed as proportion or means ± standard deviation (SD). Mean values of normally distributed continuous variables were compared using a t test. A P value <0.05 was considered statistically significant. We performed formal statistics for two comparisons: the control versus the HFD group on the same genotypes and WT versus Nrf2-null groups on the same diet.

## Results

### Nrf2 deletion causes greater liver weight gain in the Nrf2-null than in the WT mice

**Figure 1:**
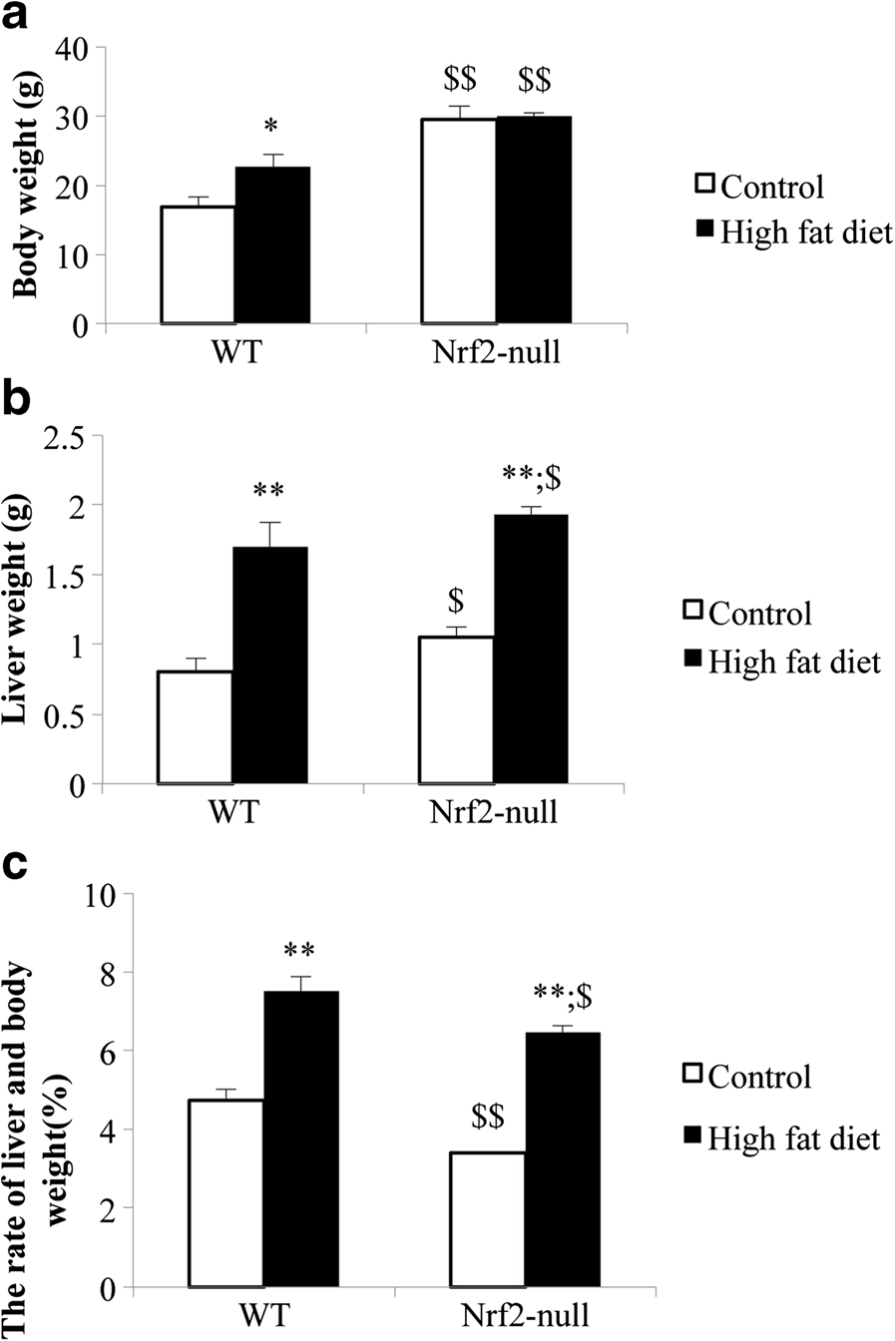
**Comparison of Nrf2 with respect to body weight, liver weight, and liver-body. a**. The influence of Nrf2 deletion on mice body weight. **b**. The influence of Nrf2 deletion on mice liver weight. **c**. The influence of Nrf2 deletion on mice rate of liver and body. Data are expressed as mean ± SD of 5 mice per group. Asterisks * represent statistical difference caused by HFD within WT and null-Nrf2 groups; dollar signs $ represent statistical difference from WT and null-Nrf2 on the same diet. 0.05 < P values < 0.01 (*, $). P values < 0.01 (**, $$). The same illustrations are used in the following figures that are applicable.

In feeding with HFD, the body weight, liver weight, and liver-body weight ratio of the WT and Nrf2-null mice were increased (Figure 1). In contrast to WT mice, the magnitude of the body weight increase in the Nrf2-null mice was lower (Figure 1a), but their liver-body weight ratio increase (2.78 ± 0.56%) were greater than those in the Nrf2-null mice ( 3.06 ± 0.28%) (Figure 1c).

### Nrf2 deletion causes histological fibrosing steatohepatitis in the mice fed the high fat diet for 4 weeks

**Figure 2:**
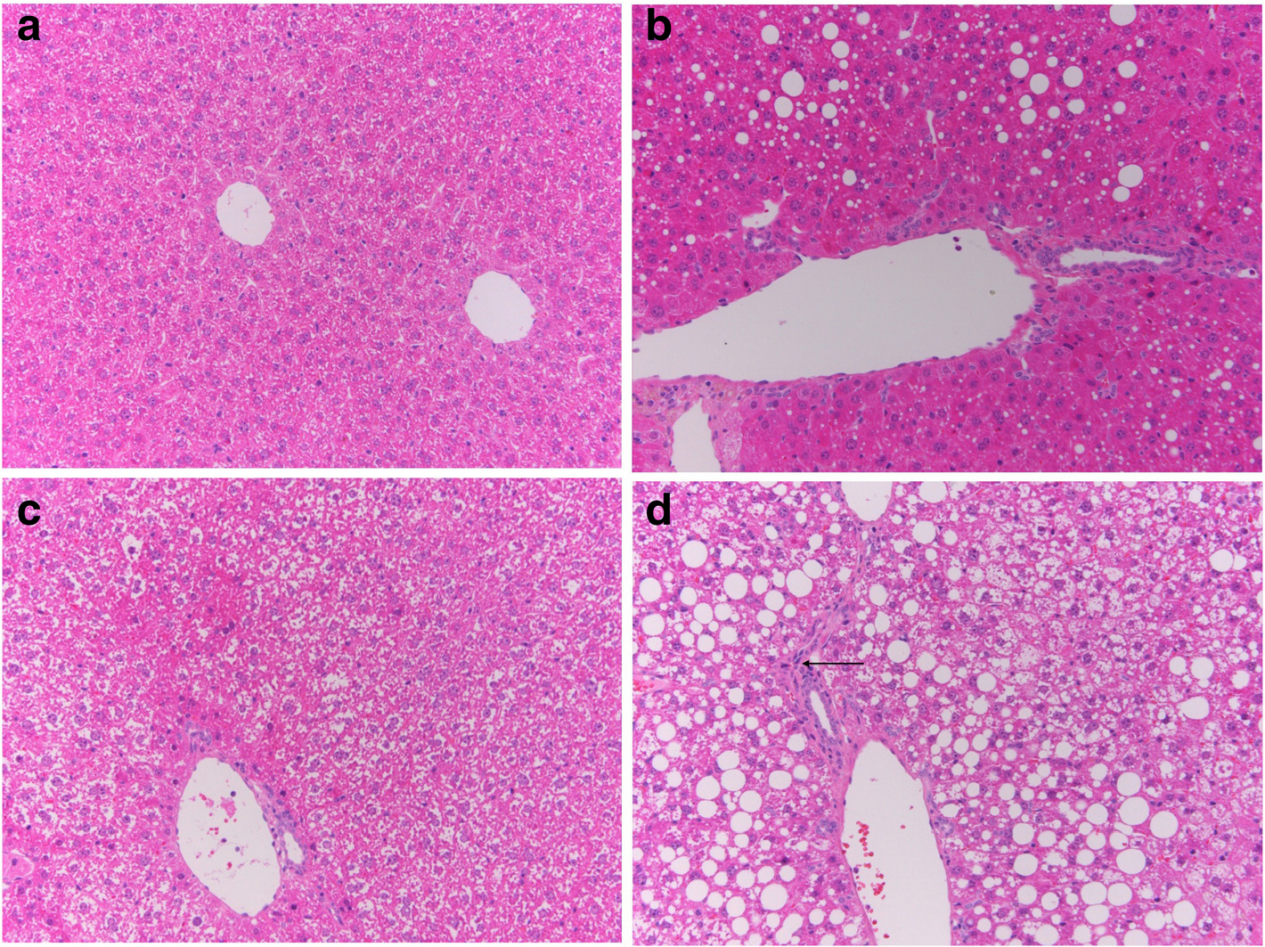
**Comparison of Nrf2 with respect to liver histopathology.** Fresh sections were stained with H & E to demonstrate lipid accumulation and flammatory cell. **a**. Wide type mouse liver induced by control diet. **b**. Wide type mouse liver induced by high fatty diet with mild hepatosteatosis consisting of mixed microvesicular and macrovesicular fat accumulation. **c**. Nrf2-null mouse liver induced by control diet with mild granular degeneration. **d**. Nrf2-null mouse liver induced by high fatty diet with severe and large scale hepatosteatosis consisting of macrovesicular fat accumulation, and mild inflammatory cell infiltration. Arrow indicates infiltrated inflamematory cells.

Histopathological examination revealed no evidence of inflammation in livers from WT-control, Nrf2-null-control, and WT-HFD mice. The livers from WT-HFD mice showed micro-lipid droplets and about 20% steatosis in the liver, whereas there was evidence of macro-lipid droplets and up to 80% steatosis in hepatocytes and inflammatory cell infiltration in the liver of Nrf2-null-HFD mice (Figure 2).

### Nrf2 deletion increases plasma levels of liver enzymes

**Figure 3:**
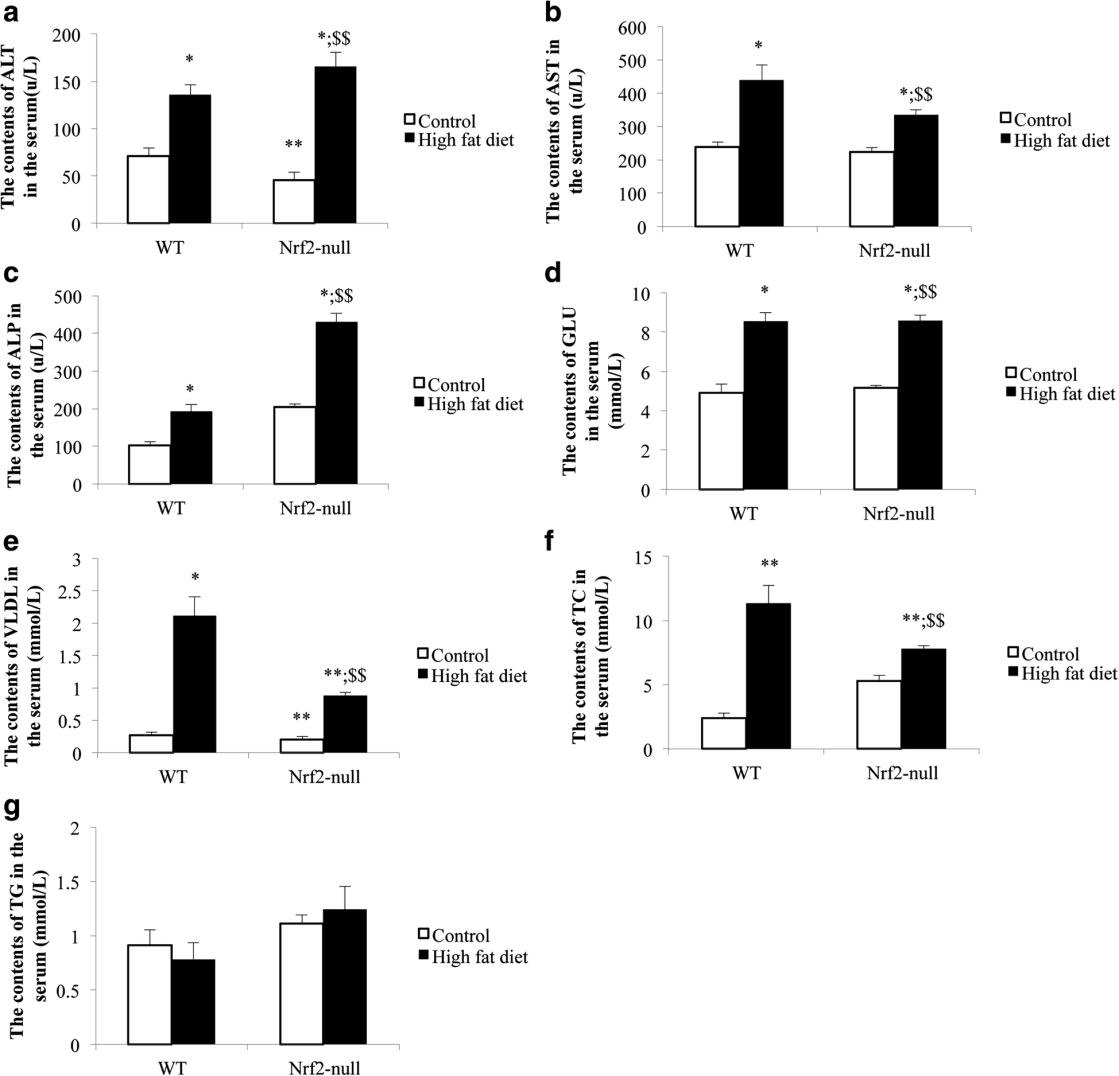
**Comparison of Nrf2 with respect to plasma laboratory tests; a.** The contents of ALT in the serum; **b**. The contents of AST in the serum; **c**. The contents of ALP in the serum; **d**. The contents of GLU in the serum; **e**. The contents of VLDL in the serum; **f**. The contents of TC in the serum; **g**. The contents of TG in the serum. ALT, alanine transaminase; AST, aspartate transminase; ALP, alkaline; GLU, glucose; VLDL, very low density lipoprotein; TC, total cholesterol; TG, triglyceride.

Plasma levels of liver enzymes and other chemicals in mice with SS or NASH are shown in Figure 3. The HFD induced the serum ALT, AST, ALP, GLU, and VLDL levels elevated in the mice of both genotypes. Nrf2 deletion caused the increased tendency of serum ALT and ALP whichenhanced, but the increased tendency of serum TC, AST, and VLDL weakened. ALT and ALP levels in Nrf2-null-HFD group (165.33 ± 14.97u/L; 430.67 ± 22.03 u/L) were significantly higher than those in WT-HFD group (135.67 ± 10.96u/L; 192.33 ± 19.14u/L). However, the serum TC, AST, and VLDL levels in Nrf2-null-HFD groups (7.81 ± 0.23 mmol/L; 336.33 ± 13.65u/L; 0.88 ± 0.45 mmol/L) were significantly lower than those in WT-HFD group (11.33 ± 1.39 mmol/L; 439.67 ± 44.16u/L; 1.12 ± 0.28 mmol/L). The serum glucose level was similar in the mice of both genotypes (8.56 ± 0.31 mmol/L; 8.53 ± 0.46 mmol/L). HFD caused the serum TG level to be elevated in Nrf2-null mice, but lowered in WT.

### Nrf2 deletion disrupts the fatty acid composition in the liver

The hepatic fatty acid composition of mice with SS or NASH is given in Table 1. The concentrations of C16: 0, C17: 0, C18: 0, C18: 1, C18: 2, C20: 1, C20: 2, C20: 3, C20: 4, and C22: 0 increased significantly in both mouse genotypes induced by HFD (p < 0.05). The concentrations of C18: 1, C20: 1, and C22: 0 in the Nrf2-null-HFD group were significantly higher than those in the WT-HFD group (p < 0.05). The HFD caused the concentrations of C12: 0, C15: 0, C18: 3, and C20: 0 to increase significantly in Nrf2-null mice (p < 0.05), but there was no significant difference in WT mice (p < 0.05). The HFD caused the concentrations of C14: 0 and C22: 6 to decrease in both mouse genotypes. Nrf2 deletion caused the reduction of C22: 6 in Nrf2-null mice compared with WT mice. The concentrations of TFA, SFA, UFA, PUFA, and MUFA were increased significantly in both mouse genotypes with HFD (p < 0.01). The concentration of UFA in the Nrf2-null-HFD group was significantly higher than in the WT-HFD group (p < 0.05), due to the significantly increased concentration of MUFA in the Nrf2-null-HFD group (p < 0.01) compared with the WT-HFD group. The HFD caused the SFA/TFA ratio and UFA/TFA ratio increase in Nrf2-null mice, but not in WT mice. The PUFA/SFA ratio decreased (p < 0.01) and the MUFA/SFA ratio increased in both mouse genotypes with HFD (p < 0.01). In contrast to the WT-HFD group, Nrf2 deletion caused the MUFA/SFA ratio to increase significantly (p < 0.01), while the PUFA/SFA ratio did not rise significantly in Nrf2-null-HFD group (p > 0.05). The HFD caused the n-6/n-6 ratio reduction in both mouse genotypes and Nrf2 deletion caused a more significant downward trend compared with WT mice.

**Table 1:** The fatty acid content and ratios in the mice liver

**Fatty acid**	**WT-control**	**WT-HFD**	**Nrf2-null-control**	**Nrf2-null-HFD**
C12: 0	0.07 ± 0.01	0.07 ± 0.01	0.07 ± 0.01	0.08 ± 0.01**
C14: 0	0.14 ± 0.01	0.13 ± 0.01	0.22 ± 0.02$$	0.19 ± 0.02$$
C15: 0	0.12 ± 0.01	0.13 ± 0.01	0.12 ± 0.01	0.14 ± 0.01*
C16: 0	5.64 ± 0.48	10.56 ± 0.78**	5.98 ± 0.54	11.59 ± 0.95**
C17: 0	0.07 ± 0.01	0.11 ± 0.01**	0.08 ± 0.01	0.12 ± 0.01**
C18: 0	4.17 ± 0.28	5.10 ± 0.41*	4.30 ± 0.37	5.46 ± 0.49*
C18: 1	7.46 ± 0.64	13.35 ± 1.16**	7.66 ± 0.67	17.40 ± 1.36**$$
c18: 2	5.13 ± 0.39	7.34 ± 0.59**	5.30 ± 0.39	8.13 ± 0.68**
C18: 3	0.03 ± 0.00	0.03 ± 0.00	0.03 ± 0.00	0.04 ± 0.00*
C20: 0	0.08 ± 0.01	0.10 ± 0.01	0.09 ± 0.01	0.15 ± 0.01**$$
C20: 1	0.08 ± 0.01	0.10 ± 0.01**	0.08 ± 0.01	0.12 ± 0.01**$
C20: 2	0.18 ± 0.01	0.31 ± 0.03**	0.19 ± 0.02	0.32 ± 0.03**
C20: 3	0.29 ± 0.03	0.38 ± 0.03**	0.30 ± 0.03	0.40 ± 0.03**
C20: 4	2.55 ± 0.21	3.26 ± 0.31**	2.74 ± 0.03	3.46 ± 0.32*
C22: 0	0.02 ± 0.00	0.03 ± 0.01*	0.03 ± 0.00	0.05 ± 0.01**$$
C22: 6	0.85 ± 0.06	0.57 ± 0.03**	0.68 ± 0.05	0.64 ± 0.05
TFA	26.88 ± 2.17	41.57 ± 3.41**	27.87 ± 2.38	48.31 ± 3.99**$
SFA	10.32 ± 0.75	16.23 ± 1.18**	10.89 ± 0.91	17.79 ± 1.44**
UFA	16.56 ± 0.91	25.34 ± 1.56**	16.98 ± 1.28	30.51 ± 2.30**$
PUFA	9.03 ± 0.36	11.90 ± 0.49**	9.25 ± 0.63	12.99 ± 0.98**
MUFA	7.53 ± 0.63	13.45 ± 1.15**	7.73 ± 0.68	17.52 ± 1.36**$$
SFA/TFA ratio (%)	38.37 ± 0.57	39.08 ± 0.37	39.24 ± 0.54	36.83 ±0.36**$$
UFA/TFA ratio (%)	61.62 ± 0.57	60.97 ± 0.37	60.93 ± 0.54	63.16 ± 0.36**$$
PUFA/SFA ratio (%)	87.69 ± 4.63	73.45 ± 3.39**	85.05 ± 3.57	73.07 ± 2.42**
MUFA/SFA ratio (%)	72.99 ± 0.83	82.80 ± 1.11**	71.02 ± 0.34	98.48 ± 0.34**$$
n-6 / n-6 ratio (%)	49.94 ± 6.59	43.49 ± 5.78	50.47 ± 2.98$$	42.65 ± 3.89*

Asterisks * represent statistical difference caused by HFD within WT and null-Nrf2 groups; dollar signs $ represent statistical difference from WT and null-Nrf2 on the same diet. 0.05 < P values < 0.01 (*, $). P values < 0.01 (**, $$).

All of the values are expressed as mean ± standard deviation (SD) (n = 20 per treatment group). TFA, total fatty acid; SFA, saturated fatty acid; UFA, unsaturated fatty acid; PUFA, polyunsaturated fatty acid; MUFA, monounsaturated fatty acid; n-6/n-6 ratio, C20: 4/C18: 2.

### Hepatic messenger RNA studies suggest that Nrf2 deletion causes NASH happened by coordinating with up-regulating IL-1β, IL-6 and TNF-α

**Figure 4:**
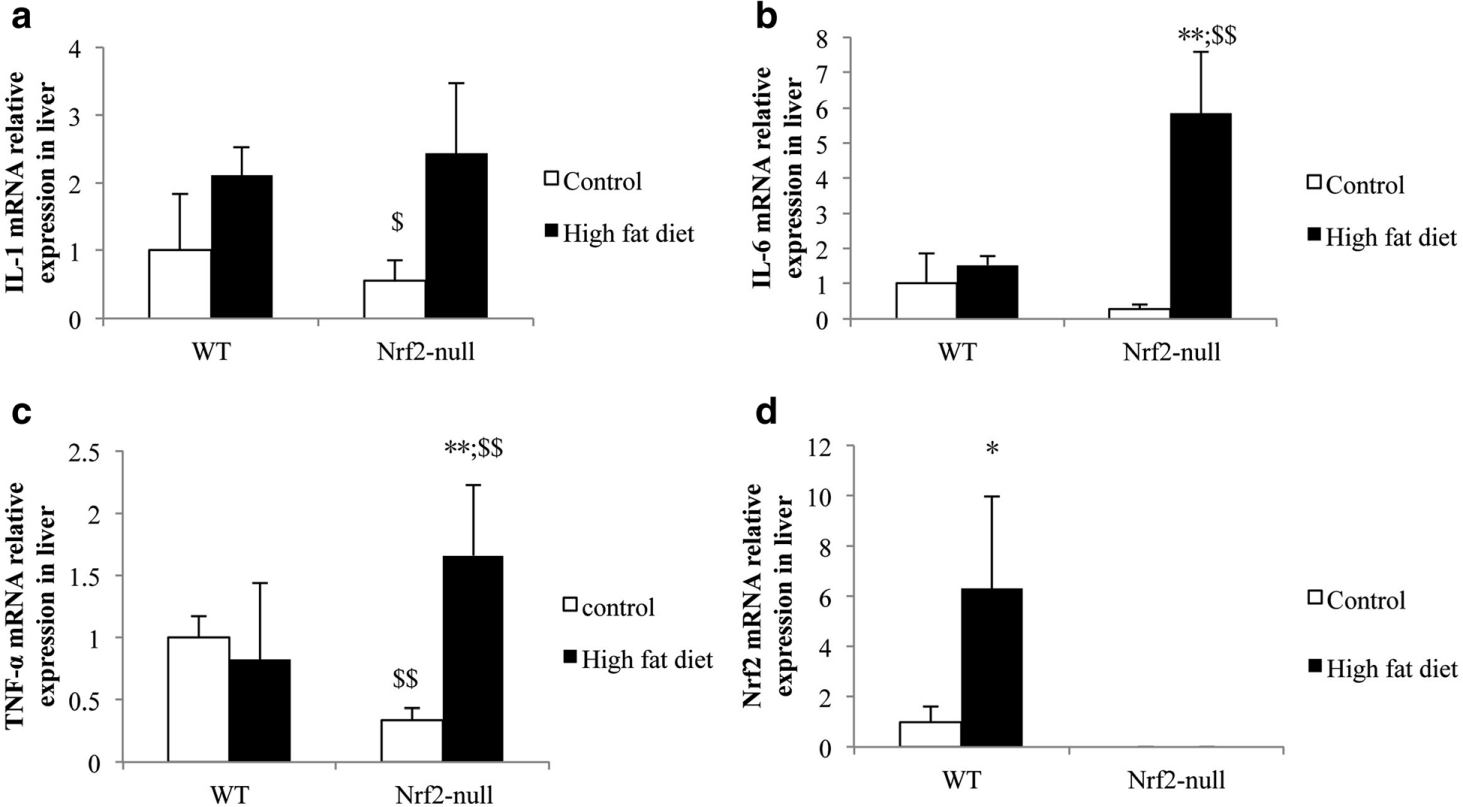
**Comparison of Nrf2 with respect to Messenger RNA expression of Nrf2, IL-1, IL-6, and TNF-α in liver. a**. The influence of Nrf2 deletion on the IL-1 mRNA expression in liver. **b**. The influence of Nrf2 deletion on IL-6 mRNA expression in liver. **c**. The influence of Nrf2 deletion on TNF-αmRNA e xpre ssion in liver. **d**. The mRNA expression in the mice of both genotypes induced by control diet and high fat diet. Nrf2, nuclear factor erythoid 2-related factor 2; IL-1, interleukin-1; IL-6, interleukin-6; TNF-α, tumor necro sis factor a lpha.

The Nrf2 deletion caused the IL-1β, IL-6, and TNF-α expression to increase in mice with HFD (Figure 4). Fed with the control diet, genotypes could influence the IL-1β, IL-6, and TNF-α expression. Nrf2 deletion mice had lower expressions of IL-1β, IL-6, and TNF-α than WT mice. The HFD-induced IL-1β expression was not significantly different in WT and Nrf2-null mice, but the gain of IL-1β expression in Nrf2-null mice was higher than in WT mice. HFD induced the IL-6 expression up-regulation in both mouse genotypes, which increased significantly in Nrf2-null mice (Figure 4b). The TNF-α expression was down-regulation in WT induced by HFD, but regulated up in Nrf2-null mice (Figure 4c). HFD induced the Nrf2 expression up-regulation significantly (Figure 4d).

## Discussion

Hepatic simple steatosis (SS) is characterized by the deposition of TG droplets in more than 5% of hepatocytes; and 10-30% of patients will develop nonalchoholic steatosis hepatitis (NASH). Our results suggest that the Nrf2 gene interacts strongly in the progression of SS to NASH induced by HFD. With feeding the HFD, Nrf2 deletion caused TG deposits in about 80% of hepatocytes, and the introduction of inflammatory cells into the liver. In contrast, the WT mice, had no inflammatory cells and had TG deposits in only 20% of hepatocytes (Figure 1). From liver pathology, it was determined that Nrf2-null mice had developed NASH, and that it had been induced by 4 weeks of HFD diet. The mechanism of hepatic accumulation of TG is attributed to both an increased uptake of fatty acid and a reduced TG secretion via VLDL [7]. HFD can cause increased uptake of fatty acid and is proven as a valuable approach to mimic NAFLD in mice [9,10]. From Figure 2, the serum TG concentration increased in Nrf2-null mice and decreased in WT mice. The serum VLDL concentration, which assists with TG secretion, was increased in both mouse genotypes; however, the increased gain in VLDL concentration in Nrf2-null mice was lower than in WT mice. This suggests that Nrf2 deletion promoted hepatic fat accumulation through a decrease of the TG secretion via VLDL. Our results reinforce the commonly held belief that Nrf2 can inhibit fat accumulation in the liver [7].

Hepatic lipid accumulation can induce insulin resistance, lipid peroxidation, hepatic cell damage, and inflammation [11]. Researchers compared the plasma fatty acid of NASH patients and control patients and found that NASH patients had a significantly higher concentration of free fatty acid (FFA), higher total saturated and monounsaturated levels in both lipid fractions and increased hexadecanoic acid and octadecenoic acid levels (C16: 0 and C18: 1) compared with control patients [12]. Similar profiles of FFA, SFA, and MUFA were found in our study. The FFA, SFA, and MUFA all increased with SS and NASH, but the TFA and MUFA concentrations in NASH were significantly higher than those in SS (Table 1). SFA (16: 0 and 18: 0) accumulation in hepatocytes could increase stress in the endoplasmic reticulum and cause hepatocyte injury [13]. Surprisingly, the excessive accumulation of SFA, eicosanoic acid and docosanoic acid (C20: 0 and C22: 0), in hepatocytes could trigger progression of SS to NASH (Table 1). Octadecenoic acid and eicosenoic acid (C18: 1 and C20: 1) concentrations in the HFD-Nrf2-null group were significantly higher than those in the HFD-WT group. It suggests that C18: 1 and C20: 1 contributed to the progression of SS to NASH. Progressive increase in MUFA is associated with the severity of the hepatic lesion [14].

PUFA forms precursors to both pro- and anti-inflammatory molecules. The balance between these mutually antagonistic compounds could determine the final outcome of disease processes [11] and play a protective role by controlling synthesis and oxidation of SFA and MUFA [15]. Our studies found that the PUFA concentrations increased in the course of SS and NASH, except docosahexanoic acid (C22: 6). HFD induced the C22: 6 decrease in the liver, which could be suppressed by Nrf2 deletion (Table 1). C22: 6 can suppress IL-1β and TNF-a production [16]. We considered that the result of increasing of C22: 6 was to resist oxidation and inflammation of the increasing SFA and MUFA in the HFD-Nrf2-null group through the suppression of IL-1β and TNF-a in contrast to the HFD-WT group. In addition, docosahexanoic acid, linolenic acid, and dihomo-linolenic acid (C22: 6, C18: 3, and C20: 3) as endogenous anti-inflammatory molecules can suppress IL-1β, TNF-a, and IL-6 production by T cells [17-22]. From Table 1, the concentration of C18: 3 in the HFD-Nrf2-null group were significantly higher than that in the HFD-WT group. Interestingly, the inflammatory cytokines IL-1β, TNF-a, and IL-6 expression increased significantly (Figure 4) and induced insulin resistance (Figure 3d) in the HFD-Nrf2-null group, compared with the HFD-WT group. Our finding suggests that IL-1β, TNF-a and IL-6 expression resulted in the significant increase of the SFA/TFA ratio and the MUFA/SFA ratio and the decrease of the PUFA/SFA ratio in the HFD-Nrf2-null group compared to the HFD-WT group (Table 1). In NASH, many pro-inflammatory cytokines are abnormal expressions [23]. The expression of TNF-a and TNF-a type 1 receptors in patients with steatohepatitis is greater than for those with steatosis alone [24,25]. The dramatic increase of TNF-a is an important function in the progression of steatosis to NASH.

The two-hit theory proposed by Day and James [26] is widely advocated as a pathologic mechanism for steatohepatitis. This theory claims that in addition to steatosis (the first hit) the development of steatohepatitis requires the presence of some other factors (the second hit). According to our results, the HFD, as the first hit, caused the superfluous TG accumulation in liver, the lipid droplets in the hepatocytes. The disrupted hepatic fatty acid promoted hepatic pro-inflammatory cytokines and pro-inflammatory molecules in the liver, leading to the second hit. Our studies suggest that the deletion of Nrf2, a lipid-metabolism regulating gene, disrupts fatty acid composition and homeostasis, causing the evolution of the “benign” SS to develop into NASH, through regulating of the MUFA/TFA ratio up and the PUFA/TFA ratio down, in conjunction with the increased hepatic concentrations of C18: 1, C20: 1, C12: 0, C22: 0, and C18: 3 in the liver. Whether the blood serum levels of fatty acids and their ratios are consistent with those in the liver needs to be determined in future studies.

## Abbreviations

NAFLD: Nonalcoholic fatty liver disease; SS: Simple steatosis; NASH: Nonalcoholic steatohepatitis; Nrf2: Nuclear erythroid 2-related factor 2; HFD: High-fat diet; wild-type: WT; ALT: Alanine aminotransferase; TC: Cholesterol; TG: Total triglyceride; AST: Aspartate transminase; ALP: Alkaline; GLU: Glucose; VLDL: Very low-density lipoprotein; C12: 0: Dodecanoic acid; C14: 0: Tetradecanoic acid; C15: 0: Pentadecanoic acid; C16: 0: Hexadecanoic acid; C17: 0: Heptadecanoic acid; C18: 0: Octadecanoic acid; C18: 1: Octadecenoic acid; C18: 2: Linoleic acid; C18: 3: Linolenic acid; C20: 0: Eicosanoic acid; C20: 1: Eicosenoic acid; C20: 2: Eicosadienoic acid; C20: 3: Dihomo-linolenic acid; C20: 4: Arachidonic acid; C22: 0: Docosanoic acid; C22:6: Docosahexanoic acid; TFA: Total fatty acid; SFA: Saturated fatty acid; UFA: Unsaturated fatty acid; PUFA: Polyunsaturated fatty acid; MUFA: Monounsaturated fatty acid; n-6/n-6 ratio: C20: 4/C18: 2; IL-1: Interleukin-1; IL-6: Interleukin-6; TNF-α: Tumor necrosis factor alpha.

## Competing interests

The authors declare that they have no competing interests.

## Authors’ contributions

CW and Pro. XZ designed the experiments and contributed to the description and writing. YC contributed to feed mice and did animal treatments, and contributed to the tissue collected. CL contributed to the fatty acids extraction and assay, YZ contributed to biochemistry assay of serum. SX contributed to the data analysis. XL contributed to the H&E staining. HL contributed to the RT-PCR assay. All authors read and approved the final manuscript.
